# Social disparities in extreme heat days across U.S. public schools

**DOI:** 10.1016/j.ssmph.2025.101835

**Published:** 2025-06-26

**Authors:** Jayajit Chakraborty, Sara Soroka

**Affiliations:** Bren School of Environmental Science & Management, University of California, 2400 Bren Hall, Santa Barbara, CA, 93106, USA

**Keywords:** School children, Extreme heat, Public school, Racial/ethnic disparities, Spatial analysis

## Abstract

Although children are highly vulnerable to higher temperatures and spend significant portions of their time at school, extreme heat events at school locations have not been adequately examined in previous research on social inequalities in the distribution and impacts of heat exposure. We address this gap by conducting the first nationwide study of sociodemographic disparities in extremely hot days at U.S. public schools. Annual frequency of extreme heat days at school locations is measured using both absolute (>90 °F) and relative (> local 95th percentile) temperature-based thresholds, and linked to race/ethnicity, socioeconomic status, and other relevant characteristics of students and schools in the conterminous U.S. Results indicate that racial/ethnic minority students and those eligible for free/reduced lunch are significantly overrepresented in schools with the highest frequency of extreme heat days (top 20 % nationally) compared to White and non-eligible students, respectively, based on the absolute temperature threshold. Similar racial/ethnic disparities are observed in the top 20 % of schools based on the relative temperature threshold, with the exception of Black and Asian students. Multivariable models that control for spatial clustering and contextual factors also reveal racial/ethnic disparities, with significantly higher frequencies of extreme heat days at schools serving Hispanic and American Indian students, regardless of the temperature threshold utilized. These results highlight the urgent need to include school children in future research on social disparities in heat exposure, conduct more detailed investigations in other regions, states, and nations, and formulate interventions and policies that provide equitable protection from extreme heat.

## Highlights

• Social disparities in school children's extreme heat exposure are understudied.
• First national study of annual frequency of extreme heat days across U.S schools.
• Racial/ethnic minority students overrepresented in schools with highest heat days.
• Disparities observed with both absolute and relative temperature thresholds.
• Schools with higher % of Hispanic and American Indian students face greater risk.

## 1 Introduction

Climate change has caused a substantial increase in the number of extreme heat days globally, adding an average of 26 extreme heat days per year, or days when temperatures are significantly hotter than what is considered normal (Arrighi et al., 2024). In the U.S., the frequency of extremely hot days has increased in 195 cities since 1970, and about 71 % of these locations now experience at least 7 additional extremely hot days each year than in 1970 (Climate Central, 2023). As the duration, frequency, and intensity of extreme heat events continue to increase ( Lu et al., 2023; Marvel et al., 2023, Meehl and Tebaldi, 2004), social disparities in the distribution and impacts of heat exposure have received considerable research and policy attention in recent years. Empirical studies on thermal inequity (Mitchell & Chakraborty, 2018) in the U.S. have found racial/ethnic minorities, lower income households, elderly populations, people with disabilities, and other vulnerable groups to be disproportionately exposed to extreme heat and its adverse health effects (Chakraborty, 2025; Chow et al., 2012; Dialesandro et al., 2021; Guo et al., 2023; Hsu et al., 2021; Johnson, 2022; Manware et al., 2022; Mitchell & Chakraborty, 2014, 2015, 2018; Renteria et al., 2022).

While excessive heat is extremely hazardous for all humans, children are particularly susceptible to its negative impacts on their health, learning, and development, and experience greater lifetime exposure to extreme heat than all previous generations, including those born just a few decades earlier (Schneider et al., 2024). Children's vulnerability to heat exposure is amplified because they are physically confined to school locations for extended periods throughout the school year. Several studies have documented that exposure to extreme heat among school children results in cognitive and developmental impairments, lower academic performance, and several other adverse health outcomes, including breathing difficulties, fatigue, and headaches (Bidassey-Manilal et al., 2016, Dapi et al., 2010, Lala and Hagishima, 2023; Porras-Salazar et al., 2018). A recent U.S. government report on heat and learning losses suggests that climate-induced temperature increases of 2 °C (3.6 °F) and 4 °C (7.2 °F) are associated with 4 % and 7 % reductions in average academic achievement per child, respectively, relative to average learning gains experienced each school year (EPA, 2023). These adverse effects are not evenly distributed, with extremely hot school days disproportionately impacting racial/ethnic minority students and accounting for about 5 % of the disparities in educational outcomes between different racial/ethnic groups (Park et al., 2020). Black, Hispanic, and socioeconomically disadvantaged students are more likely to experience the negative impacts of high-temperature events, in part, because these students attend schools reporting the lowest rates of air-conditioning availability in the U.S. (EPA, 2023).

Despite the growing concern regarding thermal inequities experienced by multiple socially disadvantaged groups, extreme heat events at school locations and related social inequalities have not been adequately examined in previous research. We address this gap by conducting the first nationwide and systematic study of sociodemographic disparities in extreme heat exposure at U.S. public schools. Specifically, the analysis seeks to answer two research questions:

1. Are racial/ethnic minority and socioeconomically deprived students significantly overrepresented in public schools burdened with the highest annual frequency of extreme heat days (top 20 % nationally)?
2. How do the racial/ethnic and socioeconomic characteristics of public schools relate to the annual frequency of extreme heat days, after accounting for spatial clustering and other relevant school characteristics?

For this study, the annual frequency of extreme heat days at school locations is measured using both absolute and relative temperature-based thresholds, as the simultaneous use of these approaches has been shown to provide more comprehensive and insightful results (Masiero et al., 2022; H. Xu & Zhang, 2022). Statistical analyses are based on bivariate comparisons and multivariable generalized estimating equations that account for the geographic clustering of public schools within U.S. counties.

## 2 Data and methods

Public schools in the conterminous U.S. (48 states and Washington D.C.) represent the unit of analysis for this study. Data on the geographic locations of public schools and their enrollment characteristics for the 2019–2020 school year were downloaded from the National Center for Education Statistics (NCES)'s Education Demographic and Geographic Estimates (EDGE) Open Data portal (NCES, 2025). The analysis excludes public schools classified as fully virtual and those designated as adult education centers.

### 2.1 Dependent variables

Previous studies of extreme heat events have utilized various heat-related indicators and threshold values, with two of the most commonly used measures being absolute temperature and relative temperature. Absolute temperature-based thresholds define extreme heat events based on fixed high-temperature values, while relative temperature thresholds consider deviations from local temperature trends to account for regional heterogeneity. While the impact of absolute extreme temperatures on human health has been studied extensively, recent studies have emphasized the need to also examine relative temperature extremes, or heat events that are highly unusual for the time of year but not necessarily extreme relative to a location's overall climate (Y. Xu et al., 2013). Accordingly, both absolute and relative temperature thresholds are used in this study to formulate two separate dependent variables that measure the annual frequency of extreme heat days at public school locations.

Census tract-level data from the Centers for Disease Control and Prevention (CDC)'s National Environmental Public Health Tracking Network (CDC, 2025a) on the annual number of extreme heat days from 2018 to 2022 were downloaded and utilized to estimate both dependent variables. Census tracts represent the smallest spatial unit for which information on extreme heat days is currently available in the U.S. The absolute threshold was defined by the annual mean number of days during which daily maximum temperatures exceeded 90 °F (°F)--the cut-off temperature used to define extreme heat by U.S. federal agencies and recent studies (Barreca, 2012; Barreca et al., 2015; Boyle et al., 2024; Masiero et al., 2022). The relative heat threshold was defined by the annual mean number of days with daily maximum temperatures exceeding the 95th percentile of the local (tract-level) temperature distribution. This measure is derived using a threshold of the 95th percentile of temperature distribution associated with each census tract, for a time period that extends from 1979 to 2019 (CDC, 2025a). This 95th percentile threshold has been employed to assess extreme heat exposure in national heat-related mapping indicators and tools such as the CDC's Environmental Justice Index (CDC and Agency for Toxic Substances Disease Registry, 2022) and Heat & Health Tracker (CDC, 2025b), as well as in published research on heatwaves (Katavoutas & Founda, 2019; Xu et al., 2013).

The annual tract-level frequency of extreme heat days was calculated by summing the number of extreme heat days and dividing by the number of years observed (i.e., five years). Tracts with missing data on extreme heat days in the Tracking Network were assigned values by interpolating the average values of their neighboring tracts, as recommended by the CDC. The school-level annual frequency of extreme heat days (2018–2022), based on both absolute and relative temperature thresholds, was derived using their values from the tract where they are located and used to represent the two dependent variables for the statistical analysis.

### 2.2 Independent variables

For each school in the conterminous U.S. from the NCES EDGE dataset, we obtained the number of enrolled students who were White, Hispanic, Black or African-American, American Indian/Alaskan Native, Asian, Hawai'ian/Other Pacific Islander, and multi-racial (two or more races). Following previous school-level studies on environmental exposure disparities in the U.S. (e.g., Chakraborty & Aun, 2023; Cheeseman et al., 2022; Collins et al., 2019; Grineski & Collins, 2018), students eligible for free or reduced lunches served as an indicator of socioeconomic deprivation, since only students from families at or below 185 % of the federal poverty level are eligible. For the school-level multivariable statistical models (Research Question 2), the total number of enrolled children was used to estimate respective percentages for these racial/ethnic and socioeconomic categories, with the White student percentage excluded from these models as the reference group.

Our analysis for Research Question 2 also included dichotomous indicators, coded as 1/0, representing the grade level of students in each school. We excluded ‘High School’ from our multivariable models as the reference group, since older children are potentially less vulnerable to extreme heat exposure than younger children (Azan et al., 2025). The NCES also classifies schools into four major designations based on their geographic locale (Geverdt & Maselli, 2024): City (territory inside a principal city and an urban area of population 50,000 or more), Suburban (territory outside a principal city and inside an urban area of population 50,000 or more), Town (territory inside an urban area of population less than 50,000), and Rural (territory outside an urban area). To account for urban-rural differences in school location, we incorporated three additional binary variables from the NCES EDGE dataset describing their geographic locale (City, Suburban, and Town) that were coded as 1/0, with schools in ‘Rural’ locales representing the reference group.

The total number of enrolled students was utilized as an additional independent variable, following previous studies on environmental exposure disparities at public schools (Chakraborty & Aun, 2023; Grineski & Collins, 2018).

### 2.3 Statistical analysis

For Research Question 1, which focuses on measuring the overrepresentation of racial/ethnic minority and socioeconomically deprived students, we first identified schools in the highest quintile, or top 20 %, for both dependent variables representing the annual frequency of extreme heat days (absolute and relative temperature thresholds). We then estimated the relative proportions of students associated with each racial/ethnic and socioeconomic deprivation category who attended schools ranked in the top 20 %, for each dependent variable. These proportions were used to calculate risk ratios, based on dividing each minority student group percentage by the percentage of White students (based on their total enrollment in conterminous US) and the percentage of free/reduced lunch eligible students by the percentage of students not eligible for free/reduced lunch, respectively. A z-test for the difference in proportions was utilized to examine if the percentages of racial/ethnic minorities and socioeconomically deprived students in the top 20 % were significantly different from the percentages of White students and those not socioeconomically deprived, respectively. These children-level bivariate comparisons encompassed a total of 47,164,243 students attending 87,215 public schools with at least one enrolled student and no missing data for race/ethnicity and free/reduced lunch eligibility.

For Research Question 2, which examines school-level statistical associations, we used multivariable generalized estimating equations (GEEs) to predict the two dependent variables representing the annual frequency of extreme heat days. GEEs are suitable here because our data are clustered and these models relax several assumptions of traditional regression (e.g., normality). GEEs assume that observations from different spatial clusters are unrelated, while observations within a cluster (e.g., schools in the same county) are related. To analyze the two dependent variables using GEEs, we selected the Tweedie distribution with logarithmic link function and an independent correlation matrix, since these model specifications provided the best statistical fit based on the quasi-likelihood under the independence model criterion (Garson, 2012). Both GEEs control for clustering based on the U.S. county where the school was located (83,662 schools in 3121 counties), with the number of schools per cluster ranging from 1 to 2137. All continuous independent variables were standardized before model entry and two-tailed p-values from the Wald Chi-square test were used to analyze the statistical significance of variable coefficients. Diagnostic testing using appropriate indicators also confirmed that our GEE models were unaffected by multicollinearity. To ensure stable percentages for all independent variables, our multivariable analysis includes 83,662 schools with more than 50 enrolled students and no missing data for any variables.

## 3 Results

**Fig. 1:**
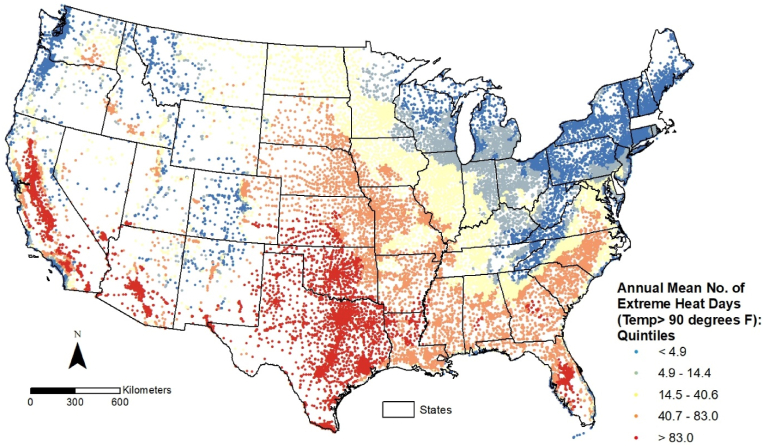
Public schools in the conterminous U.S. by annual frequency of extreme heat days (2018–2022) based on absolute high temperature threshold.

**Fig. 2:**
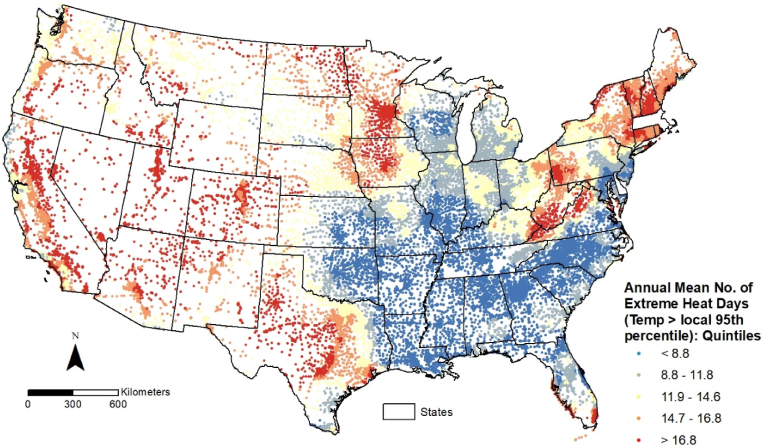
Public schools in the conterminous U.S. by annual frequency of extreme heat days (2018–2022) based on relative high temperature threshold.

School-level distributions of the annual frequency of extreme heat days, based on absolute and relative temperature thresholds, are depicted in Fig. 1, Fig. 2, respectively. Public schools in the conterminous U.S. are classified into five categories (quintiles) based on these values, with the darkest red shade representing schools ranked in the highest quintile (top 20 %) for both variables. Although most schools in California, Arizona, and Texas fall in the highest quintile on both maps, the spatial distributions of extreme heat days based on absolute (Fig. 1) and relative (Fig. 2) thresholds reveal different geographic patterns. Schools in several states of the U.S. South (e.g., Arkansas, Louisiana, Mississippi, Alabama, and Georgia) are ranked in the two highest quintiles (top 40 %) based on the absolute threshold, but fall in the lowest 20 % when the relative threshold is used. In contrast, most schools in several Northeastern (e.g., Connecticut, Maine, New Hampshire, Rhode Island, West Virginia, and Vermont) and Midwestern (e.g., Iowa and Minnesota) states are ranked in the bottom 20 % nationally based on the absolute threshold, but appear in the top 20 % based on the relative threshold.

For Research Question 1, we focused on the characteristics of enrolled students in the highest quintile of public schools (i.e., greatest annual frequency of extreme heat days) shown in Fig. 1, Fig. 2. Statistical results from these comparisons are presented in Table 1, where each minority student group's percentage (based on their group total in the conterminous U.S.) within schools ranked in the top 20 % for each dependent variable is compared to the corresponding White percentage. Similarly, the percentage of socioeconomically deprived students is compared to the corresponding percentage of those who are not socioeconomically deprived. A risk ratio (RR) greater than 1.0 indicates overrepresentation of minority student groups with respect to White students, and overrepresentation of students eligible for free/reduced lunch with respect to those not eligible, respectively.

**Table 1:** Proportion of public school students in the highest quintile (top 20 %) for annual frequency of extreme heat days, based on absolute and relative temperature-based thresholds.

	Top 20 % Schools: Absolute Threshold [Days with temp. > 90 degrees F]				Top 20 % Schools: Relative Threshold [Days with temp. > 95th percentile]			
	Percent	Risk Ratio	95 % CI of Risk Ratio	Z-test Statistic (p-value)	Percent	Risk Ratio	95 % CI of Risk Ratio	Z-test Statistic (p-value)
***Race/ethnicity:***								
Hispanic or Latino	40.18 %	2.865	[2.861, 2.868]	1680.096 (<0.0001)	27.04 %	1.456	[1.452, 1.455]	589.636 (<0.0001)
Black or African-American	17.99 %	1.283	[1.280, 1.285]	256.317 (<0.0001)	11.38 %	0.612	[0.611, 0.613]	425.688 (<0.0001)
American Indian/Alaskan Native	25.45 %	1.815	[1.806, 1.824]	234.245 (<0.0001)	28.62 %	1.539	[1.532, 1.546]	184.230 (<0.0001)
Asian	20.60 %	1.469	[1.465, 1.473]	284.764 (<0.0001)	17.30 %	0.930	[0.927, 0.933]	50.054 (<0.0001)
Hawai'ian/Other Pacific Islander	25.81 %	1.840	[1.823, 1.858]	127.430 (<0.0001)	29.26 %	1.573	[1.559, 1.586]	103.247 (<0.0001)
Two or more races	19.20 %	1.369	[1.365, 1.373]	202.905 (<0.0001)	19.02 %	1.022	[1.019, 1.025]	14.372 (<0.0001)
White [reference group]	14.02 %				18.60 %			
***Socioeconomic deprivation:***								
Free/reduced lunch eligible	26.71 %	1.451	[1.450, 1.453]	670.984 (<0.0001)	19.91 %	0.989	[0.988, 0.990]	19.697 (<0.0001)
Not eligible for free/reduced lunch [reference group]	18.40 %				20.14 %			

NOTE: N = 87,215 public schools with no missing data for all variables listed above. Risk ratios are based on dividing each minority student group percentages by the percentage of White students (based on their total enrollment in conterminous US) and percentage of free/reduced lunch eligible students by percentage of students not eligible for free/reduced lunch.

For schools in the top 20 % for annual frequency of days with temperature exceeding 90 °F, the RRs are significantly greater than 1.0 (*p* < 0.0001) for all racial/ethnic minority groups, with Hispanic students indicating the greatest RR, or overrepresentation with respect to White students. Students eligible for free/reduced lunch also reveal an RR > 1.0 (*p* < 0.0001) and significant overrepresentation compared to students who are not eligible. For schools in the top 20 % for annual frequency of heat days based on the local 95th percentile, RRs for all minority groups significantly exceed 1.0 (p < 0.0001), except for Black and Asian students (RR < 1.0), with American Indians showing the greatest overrepresentation with respect to White students. Students eligible for free/reduced lunch, however, indicate an RR < 1.0 (*p* < 0.0001) or underrepresentation compared to students who are not eligible.

Research Question 2 was analyzed using two multivariable GEE models, as depicted in Table 2. Model 1 shows that the annual frequency of extreme heat days based on the absolute temperature threshold (90 °F) is positively and significantly related to the percentage of Hispanic, Black, American Indian/Alaskan Native, and multi-racial students (*p* < 0.01), after controlling for spatial clustering and other independent variables. The annual frequency of extreme heat days is also significantly greater in schools with higher enrollment, those serving younger students (i.e., primary and middle schools), and those in predominantly rural areas (p < 0.001).

**Table 2:** Multivariable generalized estimating equations (GEEs) for predicting annual frequency of extreme heat days, based on absolute and relative temperature-based thresholds.

	Model 1: Absolute Threshold [Days with temp. > 90 degrees F]				Model 2: Relative Threshold [Days with temp. > 95th percentile]			
	Beta	Lower 95 % CI	Upper 95 % CI	P-value	Beta	Lower 95 % CI	Upper 95 % CI	P-value
Total number of enrolled students	0.089	0.067	0.111	<0.001	−0.029	−0.040	−0.019	<0.001
% Hispanic or Latino	0.512	0.440	0.583	<0.001	0.104	0.083	0.125	<0.001
% Black or African-American	0.210	0.159	0.260	<0.001	−0.117	−0.150	−0.083	<0.001
% Asian	−0.003	−0.074	0.068	0.934	−0.004	−0.019	0.012	0.654
% American Indian/Alaskan Native	0.088	0.063	0.112	<0.001	0.019	0.008	0.029	<0.001
% Hawai'ian/Other Pacific Islander	0.012	−0.024	0.048	0.514	0.027	0.012	0.041	<0.001
% Two or more races	0.070	0.021	0.118	0.005	−0.011	−0.025	0.004	0.166
% Free or reduced lunch eligible	−0.064	−0.246	0.117	0.485	−0.058	−0.074	−0.042	<0.001
*School Level*:								
Prekindergarten	0.121	0.009	0.232	0.034	−0.022	−0.055	0.011	<0.001
Elementary	0.106	0.052	0.161	<0.001	−0.020	−0.034	−0.006	<0.001
Middle	0.085	0.048	0.123	<0.001	−0.036	−0.047	−0.026	<0.001
Ungraded/Other	0.202	0.137	0.266	<0.001	0.041	0.016	0.067	0.891
*Locale:*								
City	−0.426	−0.617	−0.235	<0.001	0.143	0.110	0.177	<0.001
Suburb	−0.538	−0.653	−0.423	<0.001	0.077	0.045	0.110	<0.001
Town	−0.118	−0.161	−0.076	<0.001	0.041	0.023	0.060	<0.001
Intercept	3.884	3.820	3.947	<0.001	2.465	2.440	2.490	<0.001

NOTE: Reference groups are % White for race/ethnicity, High School for school level, and Rural for locale. GEEs for both models are based on a Tweedie distribution with logarithmic link function and independent correlation matrix. All continuous independent variables were standardized before model entry. P-values are based on the Wald Chi-Square test. N = 83,662 public schools with more than 50 enrolled students and no missing data for all variables.

Model 2 indicates that extreme heat days based on the relative temperature threshold (local 95th percentile) are positively and significantly related to the percentage of Hispanic, American Indian/Alaskan Native, and Hawai'ian/Pacific Islander students (*p* < 0.001), but negatively associated with the proportion of Black and reduced/free lunch eligible students (*p* < 0.001). In contrast to the results noted for Model 1, the use of the relative temperature threshold results in observations of significantly higher frequencies of extreme heat days in lower enrollment schools, those serving older students (i.e., high schools), and schools in non-rural locations (*p* < 0.001).

## 4 Discussion

This study sought to investigate spatial and social disparities in exposure to extreme heat days across public schools and enrolled students in the conterminous U.S. Research Question 1 examined whether racial/ethnic minority and socioeconomically deprived students are overrepresented in schools with the highest annual frequency of extreme heat days, compared to students who are White and not socioeconomically deprived, respectively. Results indicate that students from all racial/ethnic minority groups and those eligible for free/reduced lunch are significantly overrepresented in schools with the highest frequency (top 20 % nationally), based on the absolute (90 °F) temperature threshold. Similar racial/ethnic disparities were observed in the top 20 % of schools based on the relative (local 95th percentile) threshold, with the exception of Black and Asian students.

Research Question 2 focused on analyzing disparities in annual frequency of extreme heat days based on sociodemographic characteristics and grade level of schools, after adjusting for enrollment size, urban/rural location, and spatial clustering of schools at the county level. Multivariable models indicated increased frequency of heat days in schools with higher percentages of Hispanic, Black, American Indian, and multi-racial students, based on the absolute temperature threshold, and higher percentages of Hispanic, American Indian, and Pacific Islander students based on the relative threshold. While schools with greater proportions of Hispanic and Native American students are burdened by significantly greater extreme heat days based on both thresholds, the percentages of Black and free/reduced lunch students indicate negative associations with the frequency of heat days when the relative threshold is used.

These differences in model results associated with absolute vs. relative thresholds can be explained, in part, by a higher prevalence of Black and socioeconomically deprived children in schools located in the U.S. South, where summer temperatures have exceeded 90 °F frequently in recent years (Paschal, 2023), but days with temperatures rising above the high local averages or 95th percentile threshold are relatively less common. The negative relationship between extreme heat days and the percentages of Black or free/reduced lunch students also reflects lower proportions of both these student groups across schools located in multiple Northeastern and Midwestern states that experienced a higher frequency of extreme heat days based on the 95th percentile threshold, but a lower frequency of heat days based on the 90 °F threshold. However, a majority of public schools in Western and Southwestern states are characterized by higher proportions of Hispanic and American Indian students, as well as a greater number of extreme heat days annually based on both absolute and relative temperature thresholds.

The results from our study are consistent with previous national-scale research that found significant racial/ethnic disparities in the spatial distribution of air and noise pollution across U.S. schools and children (Cheeseman et al., 2022; Collins et al., 2019; Grineski and Collins, 2018, Grineski and Collins, 2019; Kingsley et al., 2014). Since these studies did not examine thermal burdens faced by school children, our findings emphasize the need for additional research on the negative impacts of extreme heat on racial/ethnic minority students, especially in public schools characterized by higher Hispanic and Native American enrollment.

### 4.1 Study limitations and recommendations

Although this study represents an important first step in analyzing social disparities in extreme heat exposure for school children, it is important to consider three limitations. First, our measurement of the dependent variable associated with both absolute and relative temperature thresholds is based on data from the CDC's National Environmental Public Health Tracking Network that covers all 12 months of each year (annual totals), which may not align with a school's academic calendar or when students are actually present at school locations. Although data on monthly or weekly frequencies of extreme heat days are not published by the CDC at the census tract level, future research should explore additional data sources that allow the exclusion of specific time periods such as months or weeks that coincide with public school closures.

Second, while our study demonstrates how school-level sociodemographic disparities are sensitive to the choice of high temperature threshold (absolute vs. relative), we are unable to make specific recommendations regarding which extreme heat measure is more appropriate or useful than the other. To address this limitation, we recommend more research and scholarship that provides detailed and systematic knowledge on the environmental, health, and policy implications of selecting each high temperature threshold with respect to children's well-being and school performance, especially for marginalized and vulnerable students facing greater extreme heat exposure.

Third, our analysis did not consider heat mitigation factors such as the presence of air conditioning or tree canopy cover at school locations because no data source currently provides high-resolution and nationally consistent data on these indicators that can be linked to all public schools in conterminous U.S. Future work should examine heat reduction and mitigation strategies adopted by schools experiencing a higher frequency of extremely hot days, as well as barriers and challenges faced by public schools to provide adequate and equitable protection from extreme heat events.

## 5 Conclusion

This study contributes to the growing research literature on social disparities in the distribution of extreme heat through a national-scale analysis of public school children—a particularly vulnerable subpopulation that has not been examined in previous thermal inequity research. Unlike previous studies on this topic that relied on a single indicator of extreme heat exposure, heat burdens were measured using two variables that encompass both absolute and relative temperature-based thresholds. Overall, our results reveal significant racial/ethnic disparities in the distribution of extreme heat days across U.S. public schools, even after controlling for spatial clustering and relevant school characteristics. Using both absolute and relative thresholds, we find that schools experiencing a higher frequency of extreme heat days contain disproportionately greater proportions of Hispanic and American Indian students.

Our results also indicate that school-level sociodemographic disparities are influenced by the threshold used for defining extremely hot days. Specifically, Black students and high-enrollment rural schools are overrepresented in areas facing excessive extreme heat days based on the absolute temperature threshold, but underrepresented in areas with excessive heat days based on the relative threshold. These findings underscore the necessity for more data-driven and evidence-based research on thermal inequities experienced by minority, underserved, and vulnerable school children using multiple indicators of extreme heat, heatwave occurrence, and their potential impacts.

The racial/ethnic disparities associated with extreme heat days we found in this study have significant policy implications, as children's exposure to higher temperatures has been linked to school absences, poorer academic performance, and diminished achievement. These adverse outcomes, in combination with increased probabilities of various health risks, potentially limit students' present and future success. As the intensity and frequency of heatwaves and extreme heat days continue to increase, it is imperative to formulate and implement appropriate mitigation strategies that focus on reducing children's heat exposure. This is especially necessary among schools attended by higher percentages of racial/ethnic minority students, as these schools are often additionally burdened with other challenges, including limited financial support, smaller annual budgets, and teacher shortages (Chakraborty, 2022). These challenges are likely to prevent these schools from accessing and implementing proper protections for their students, which can include air conditioning, green spaces, and heat-resilient infrastructure. These interventions would require concerted and sustained investment, as well as multisector collaboration between educators, public health professionals, policymakers, climate scientists, and various other stakeholders.

## CRediT authorship contribution statement

**Jayajit Chakraborty:** Writing – review & editing, Writing – original draft, Software, Project administration, Methodology, Formal analysis, Data curation, Conceptualization. **Sara Soroka:** Writing – review & editing, Methodology, Formal analysis.

## Ethics in publishing statement

I testify on behalf of all co-authors that our article submitted followed ethical principles in publishing.

This research presents an accurate account of the work performed, all data presented are accurate and methodologies detailed enough to permit others to replicate the work.

This manuscript represents entirely original works and or if work and/or words of others have been used, that this has been appropriately cited or quoted and permission has been obtained where necessary.

This material has not been published in whole or in part elsewhere.

The manuscript is not currently being considered for publication in another journal.

That generative AI and AI-assisted technologies have not been utilized in the writing process or if used, disclosed in the manuscript the use of AI and AI-assisted technologies and a statement will appear in the published work.

That generative AI and AI-assisted technologies have not been used to create or alter images unless specifically used as part of the research design where such use must be described in a reproducible manner in the methods section.

All authors have been personally and actively involved in substantive work leading to the manuscript and will hold themselves jointly and individually responsible for its content.

## Funding

This research did not receive any specific grant from funding agencies in the public, commercial, or not-for-profit sectors.

## Declaration of competing interest

The authors declare that they have no known competing financial interests or personal relationships that could have appeared to influence the work reported in this paper.

## Data Availability

Data will be made available on request.

## References

[bib1] Arrighi J., Otto F.E.L., Marghidan C.P., Philip S., Singh R., Vahlberg M., Giguere J., Pershing A.J., Tannenbaum A., Veitch A. (2024). Climate change and the escalation of global extreme heat: Assessing and addressing the risks. https://assets.ctfassets.net/cxgxgstp8r5d/5sjPWtBWuPk56xVZKuuL3g/710d0a89e6eb859b1dc0417cb718dea8/Climate_Central_Climate_Change_and_the_Escalation_of_Global_Extreme_Heat.pdf.

[bib2] Azan A., Nyimbili S., Babayode O.O., Bershteyn A. (2025). Exceeding the limits of paediatric heat stress tolerance: The risk of losing a generation to climate inaction. BMJ Paediatrics Open.

[bib3] Barreca A. (2012). Climate change, humidity, and mortality in the United States. Journal of Environmental Economics and Management.

[bib4] Barreca A., Clay K., Deschênes O., Greenstone M., Shapiro J.S. (2015). Convergence in adaptation to climate change: Evidence from high temperatures and mortality, 1900–2004. The American Economic Review.

[bib5] Bidassey-Manilal S., Wright C.Y., Engelbrecht J.C., Albers P.N., Garland R.M., Matooane M. (2016). Students' perceived heat-health symptoms increased with warmer classroom temperatures. International Journal of Environmental Research and Public Health.

[bib6] Boyle C.F., Clark C.E., Horn D.P., Jaroscak J.V., Kreiser M., Lawhorn J.M., Lee E.A., Perl L., Sekar K., Sheikh H.Z., Webster E.M. (2024). Emergency response to extreme heat: Federal financial assistance and considerations for congress (No. R46873). https://www.congress.gov/crs-product/R46873.

[bib8] CDC and Agency for Toxic Substances Disease Registry (2022). Environmental Justice Index.

[bib7] Centers for Disease Control and Prevention (CDC) National Environmental Public Health Tracking Network Data Explorer. https://ephtracking.cdc.gov/DataExplorer.

[bib9] CDC (2025). Heat and Health Tracker. https://ephtracking.cdc.gov/Applications/heatTracker/.

[bib10] Chakraborty J. (2022). Children's exposure to vehicular pollution: Environmental injustice in Texas, USA. Environmental Research.

[bib11] Chakraborty J. (2025). Heatwave frequency and disability status: Thermal inequities in the U.S. South. Disability and Health Journal.

[bib12] Chakraborty J., Aun J.J. (2023). Social inequities in exposure to traffic-related air and noise pollution at public schools in Texas. International Journal of Environmental Research and Public Health.

[bib13] Cheeseman M.J., Ford B., Anenberg S.C., Cooper M.J., Fischer E.V., Hammer M.S., Magzamen S., Martin R.V., van Donkelaar A., Volckens J., Pierce J.R. (2022). Disparities in air pollutants across racial, ethnic, and poverty groups at US public schools. GeoHealth.

[bib14] Chow W.T.L., Chuang W.-C., and P.G. (2012). Vulnerability to extreme heat in metropolitan phoenix: Spatial, temporal, and demographic dimensions. The Professional Geographer.

[bib15] Climate Central (2023). More extremely hot days | climate central. https://www.climatecentral.org/climate-matters/more-extremely-hot-days-2023.

[bib16] Collins T.W., Grineski S.E., Nadybal S. (2019). Social disparities in exposure to noise at public schools in the contiguous United States. Environmental Research.

[bib17] Dapi L.N., Rocklöv J., Nguefack-Tsague G., Tetanye E., Kjellstrom T. (2010). Heat impact on schoolchildren in Cameroon, Africa: Potential health threat from climate change. Global Health Action.

[bib18] Dialesandro J., Brazil N., Wheeler S., Abunnasr Y. (2021). Dimensions of thermal inequity: Neighborhood social demographics and urban heat in the Southwestern U.S. International Journal of Environmental Research and Public Health.

[bib19] EPA (2023). https://primarysources.brillonline.com/browse/climate-change-and-law-collection/climate-change-and-childrens-health-and-wellbeing-in-the-united-states;cccc016620230788.

[bib20] Garson G.D. (2012).

[bib21] Geverdt D., Maselli A. (2024). https://nces.ed.gov/programs/edge/Geographic/LocaleBoundaries.

[bib22] Grineski S.E., Collins T.W. (2018). Geographic and social disparities in exposure to air neurotoxicants at U.S. public schools. Environmental Research.

[bib23] Grineski S.E., Collins T.W. (2019). Lifetime cancer risks from hazardous air pollutants in US public school districts. Journal of Epidemiology & Community Health.

[bib24] Guo Y., Chen P., Xie Y., Wang Y., Mu Y., Zhou R., Niu Y., Shi X., Zhu J., Liang J., Liu Q. (2023). Association of daytime-only, nighttime-only, and compound heat waves with preterm birth by urban-rural area and regional socioeconomic status in China. JAMA Network Open.

[bib25] Hsu A., Sheriff G., Chakraborty T., Manya D. (2021). Disproportionate exposure to urban heat island intensity across major US cities. Nature Communications.

[bib26] Johnson D.P. (2022). Population-based disparities in U.S. urban heat exposure from 2003 to 2018. International Journal of Environmental Research and Public Health.

[bib27] Katavoutas G., Founda D. (2019). Response of urban heat stress to heat waves in Athens (1960–2017). Atmosphere.

[bib28] Kingsley S.L., Eliot M.N., Carlson L., Finn J., MacIntosh D.L., Suh H.H., Wellenius G.A. (2014). Proximity of US schools to major roadways: A nationwide assessment. Journal of Exposure Science and Environmental Epidemiology.

[bib29] Lala B., Hagishima A. (2023). Impact of escalating heat waves on students' well-being and overall health: A survey of primary school teachers. Climate.

[bib30] Lu R., Xu K., Chen R., Chen W., Li F., Lv C. (2023). Heat waves in summer 2022 and increasing concern regarding heat waves in general. Atmospheric and Oceanic Science Letters.

[bib31] Manware M., Dubrow R., Carrión D., Ma Y., Chen K. (2022). Residential and race/ethnicity disparities in heat vulnerability in the United States. GeoHealth.

[bib32] Marvel K., Su W., Delgado R., Aarons S., Chatterjee A., Garcia M.E., Hausfather Z., Hayhoe K., Hence D.A., Jewett E.B., Robel A., Singh D., Tripati A., Vose R.S., Crimmins A.R., Avery C.W., Easterling D.R., Kunkel K.E., Stewart B.C., Maycock T.K. (2023). Fifth national climate assessment.

[bib33] Masiero G., Mazzonna F., Santarossa M. (2022). The effect of absolute versus relative temperature on health and the role of social care. Health Economics.

[bib34] Meehl G.A., Tebaldi C. (2004). More intense, more frequent, and longer lasting heat waves in the 21st century. Science.

[bib35] Mitchell B.C., Chakraborty J. (2014). Urban heat and climate justice: A landscape of thermal inequity in pinellas county, Florida. Geographical Review.

[bib36] Mitchell B.C., Chakraborty J. (2015). Landscapes of thermal inequity: Disproportionate exposure to urban heat in the three largest US cities. Environmental Research Letters.

[bib37] Mitchell B.C., Chakraborty J. (2018).

[bib38] National Center for Education Statistics (NCES) NCES Education Demographic and Geographic Estimates (EDGE) Open Data Portal. https://data-nces.opendata.arcgis.com/.

[bib45] Park R.J., Goodman J., Hurwitz M., Smith J. (2020). Heat and Learning. American Economic Journal: Economic Policy.

[bib39] Paschal O. (2023). https://www.theatlantic.com/science/archive/2023/07/south-extreme-heat-wave-texas-climate-change/674598/.

[bib40] Porras-Salazar J.A., Wyon D.P., Piderit-Moreno B., Contreras-Espinoza S., Wargocki P. (2018). Reducing classroom temperature in a tropical climate improved the thermal comfort and the performance of elementary school pupils. Indoor Air.

[bib41] Renteria R., Grineski S., Collins T., Flores A., Trego S. (2022). Social disparities in neighborhood heat in the Northeast United States. Environmental Research.

[bib42] Schneider A., Shoemaker DeMio P., Gibbs H., Partelow L. (2024). https://www.americanprogress.org/article/protecting-children-from-extreme-heat-is-critical-for-their-health-learning-and-development/.

[bib43] Xu Y., Dadvand P., Barrera-Gómez J., Sartini C., Marí-Dell’Olmo M., Borrell C., Medina-Ramón M., Sunyer J., Basagaña X. (2013). Differences on the effect of heat waves on mortality by sociodemographic and urban landscape characteristics. Journal of Epidemiology & Community Health.

[bib44] Xu H., Zhang G. (2022). Comparison of relative and absolute heatwaves in Eastern China: Observations, simulations and future projections. Atmosphere.

